# A multimodal dataset for process monitoring and anomaly detection in industrial CNC milling

**DOI:** 10.1016/j.dib.2025.112207

**Published:** 2025-10-22

**Authors:** Robin Ströbel, Maximilian Kuck, Florian Oexle, Hafez Kader, Alexander Puchta, Benjamin Noack, Jürgen Fleischer

**Affiliations:** a wbk Institute of Production Science, Karlsruhe Institute of Technology (KIT), 76131 Karlsruhe, Germany; b AMS–Autonomous Multisensor Systems, Otto von Guericke University Magdeburg, 39106 Magdeburg, Germany

**Keywords:** Process monitoring, Anomaly detection, Milling, Machine tool, CNC

## Abstract

During the fourth industrial revolution, agile production methods have gained increasing importance to meet the growing demand for product individualization. Conventional process monitoring systems, which predominantly rely on static, statistically based approaches, are insufficient for the requirements of flexible manufacturing environments. Although numerous research initiatives have proposed machine learning (ML) based solutions for agile process monitoring, widespread adoption in industrial practice has not yet been achieved. This is partly due to a lack of system comparability and insufficient validation under realistic production conditions.

To address this, a comprehensive dataset was recorded on a DMC 60 H three-axis milling machine by Deckel Maho. The dataset comprises multiple signals from a Siemens SINUMERIK 840D controller, recorded at 500 Hz via a Siemens SINUMERIK Edge. These were synchronized with force and acceleration data (sampled at 10 kHz) captured via a force measurement platform and acceleration sensors. A total of 32 experiments were conducted (15 with 8 distinct anomaly types), resulting in nearly 8 million data points per signal and six hours of process data.

Key features include:

• Realistic workpiece geometries (thermoforming molds, injection molds, pump impellers) representing diverse milling scenarios.

• Multiple anomalies (e.g., tool wear, chatter, material defects) to enable targeted validation.

• Full reproducibility through provided NC codes, CAD models, and raw/processed data formats (.json, .mat, .csv, .stp, .nc).

The dataset is intended to serve as a benchmark for industrial stakeholders to evaluate monitoring systems, as well as providing researchers with a secondary resource for benchmarking and optimizing ML-based approaches. By fostering comparability, it aims to bridge the gap between theoretical frameworks and industrial application.

Specifications table

**Table:** 

Subject	Engineering & Materials science
Specific subject area	Validation of intelligent process monitoring systems on a CNC milling machine
Type of data	Raw Data: - JSON (.json) - MATLAB timetable (.mat) Processed: - CSV (.csv) - MATLAB timetable (.mat) - NC—Code (.nc) - CAD-Models (.stp)
Data collection	Approximately six hours of milling processes were performed on a three-axis milling machine (DMC 60 H, Deckel Maho). Controller-side process signals - such as axis positions, spindle speed, motor current, and torque - were acquired from the Siemens SINUMERIK 840D CNC system via the SINUMERIK Edge app *Analyze MyWorkpiece/Capture*, which enables high-frequency data export at 500 Hz. Additional force measurements were recorded with a force measurement platform (Kistler Type 9255C), and accelerations were captured using a sensor mounted on the main spindle (PCB Type 356A33). The force platform signals were amplified using a charge amplifier (Kistler Type 5015A1000 K) before being acquired at a sampling rate of 10 kHz via a data acquisition card (Data Translation DT9836; referred to as DAC). The acceleration sensor was connected to the same DAC via a Kistler coupler (type 5122).
Data source location	wbk – Institut für Produktionstechnik, Karlsruher Institut für Technologie (KIT)
Data accessibility	Repository name: Multimodal Dataset for Process Monitoring and Anomaly Detection in Industrial CNC Milling Data identification number: 10.35097/hvvwn1kfwf7qt48z Direct URL to data: https://publikationen.bibliothek.kit.edu/1000182633
Related research article	none

## 1 Value of the Data

• This dataset was created with the aim of providing a broad range of practice-oriented production data. The methodological framework used was Design Science Research [7] within which a variety of scientific methods were employed to develop a rigorous and application-oriented experimental design. During the experiments, extensive time series data of various process signals were recorded.
• A key feature of the dataset is the integration of eight different anomalies into the experiment series. These were methodically selected to realistically reflect typical challenges in milling operations.
• The dataset is highly representative of industrial practice: real components were machined, covering numerous aspects of varying production conditions through the deliberate selection of different part types. Three representative components were manufactured to reflect flexible production environments: a thermoforming mold with simple, straight-lined geometry; a complex injection mold with a multi-stage machining process; and twelve impellers with varying blade geometries.
• All experiments are fully reproducible and transparently documented, enabling standardized and quantifiable comparisons between different process monitoring systems. The dataset includes not only raw process signals but also the corresponding part models and all associated NC codes.
• The dataset was developed with a focus on generalizability. It includes experiments involving different parts, materials, and process parameters. Its modular structure allows targeted analysis of specific aspects of agile manufacturing. An exemplary data evaluation is included, and a supplementary guide supports the application of the dataset in various research and industrial scenarios.

## 2 Background

As product individualization continues to progress during the fourth industrial revolution, conventional statistical approaches to process monitoring are increasingly reaching their limits. In response, numerous research initiatives are developing agile and intelligent process monitoring strategies. However, these approaches are often validated using datasets that vary significantly in structure and scope, and in many cases, lack detailed documentation or real-world applicability [4,5,18]. This diversity in validation conditions makes it difficult to assess the actual performance of such systems in a comparable and practical manner. For industrial users, this can limit transparency and complicate the evaluation of potential benefits for real-world implementation.

The dataset presented aims to bridge the gap between academic research and industrial application. It is a transparent, practice-oriented, and methodologically sound dataset based on primary experimental data, enabling standardized validation and evaluation of process monitoring systems. Such benchmark datasets are already common in the field of ML - for example, in the comparison of large language models [16,19].

The underlying methodological framework is Design Science Research [7,14], which ensured the systematic application of scientific methods throughout all development steps. A morphological box was used to design parts that differ in five central parameters and represent real-world manufacturing scenarios. Key influencing factors in milling processes were identified and deliberately integrated into the part geometries. Additionally, quality-relevant variables were analyzed, and the most significant ones were introduced as intentional anomalies during the experiments. Throughout the development, key quality criteria for datasets were rigorously followed.

## 3 Data Description

**Fig. 1:**
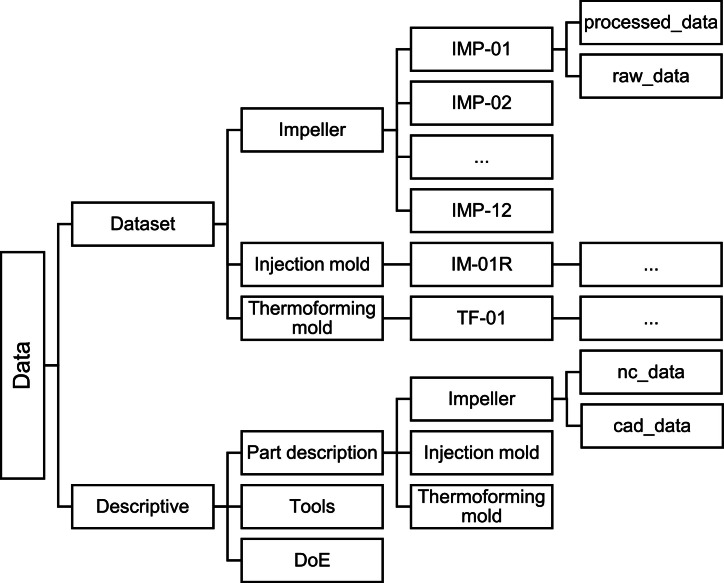
Structure of the dataset.

The following section describes the structure and content of the dataset as seen in Fig. 1. The folder structure distinguishes between the measurement data located in the “*dataset*” directory and the corresponding experimental documentation found in the “*descriptive*” folder.

In the “*dataset*” folder, the data are organized by the three part types: impellers (IMP), injection molds (IM), and thermoforming molds (TF). Each experiment conducted is stored in a separate folder named according to a consistent scheme. The impeller folder also includes a dataset for the base contour (IMP-BASE) required for the machining of the impellers. The first label indicates the part type, the second the experiment number (with identical toolpath strategy), and the third indicates the anomaly number, if applicable. For example, “IM-01-A02” refers to the first geometry of the injection mold part with the second anomaly in this experiment. A detailed mapping of all experiments is included in the Design of Experiments (DoE), while a summary is presented in Table 1. Each experiment folder follows the same internal structure:

- The *raw_data* folder contains the unprocessed signals from the data acquisition systems. The SINUMERIK Edge recordings (500 Hz) are included in JSON format. Force and acceleration data were recorded at a sampling rate of 10 kHz and are stored as MATLAB timetable files (.mat).
- The *processed_data* folder contains preprocessed CSV files derived from the Edge recordings *(header.csv, hfdata.csv, hfblockevent.csv).* This folder also includes a MATLAB timetable file in which all process signals have been synchronized and combined. Additionally, a MATLAB figure (Sync_Plot.fig) is included to visualize and confirm the successful synchronization between the DAC and SINUMERIK signals.

**Table 1:** Overview of experiments with tool, material, anomaly type, and process duration.^a^

Component	Trial	Anomalies	Tool	Material	Duration [s]
Thermoforming mold	TF-01	-	HSS 20 mm	Al 2007 T4	240.35
	TF-02	-	HSS 20 mm	Al 2007 T4	246.27
	TF-03	-	HSS 20 mm	Al 2007 T4	246.55
	TF-01-A01	Increased feed rate	HSS 20 mm	Al 2007 T4	201.16
	TF-02-A01	built-up edge	HSS 20 mm	Al 2007 T4	491.50
	TF-03-A01	Overload	HSS 20 mm	Al 2007 T4	146.10
	TF-03-A02	Overload	HSS 20 mm	Al 2007 T4	99.10
Injection mold	IM-01R	-	SCEM 10 mm	S235JR	1190.92
	IM-01R-A01	Tool wear (*t* = 1709.93 s) & blowhole (depth: 1 mm)	SCEM 10 mm	S235JR	1191.67
	IM-01R-A02	Tool wear (*t* = 3419.88 s) & blowhole (depth: 5 mm)	SCEM 10 mm	S235JR	1191.61
	IM-01R-A03	Tool wear (*t* = 5129.83 s)	SCEM 10 mm	S235JR	1190.98
	IM-01R-A04	Tool wear (*t* = 6893.77 s)	SCEM 10 mm	S235JR	1191.55
	IM-01R-A05	Tool wear (*t* = 8549.71 s)	SCEM 10 mm	S235JR	1191.43
	IM-01F	-	SCEM 10 mm	S235JR	592.98
	IM-01F-A01	Irregular Stock Allowance	SCEM 10 mm	S235JR	592.98
	IM-02F-A01	Chatter	SCEM 10 mm	S235JR	303.95
Impeller	IMP-BASE	-	SCEM 5 mm	POM-C	758.10
	IMP-01	-	SCEM 5 mm	POM-C	325.89
	IMP-02	-	SCEM 5 mm	POM-C	335.58
	IMP-03	-	SCEM 5 mm	POM-C	276.20
	IMP-04	-	SCEM 5 mm	POM-C	244.04
	IMP-05	-	SCEM 5 mm	POM-C	291.38
	IMP-06	-	SCEM 5 mm	POM-C	251.82
	IMP-07	-	SCEM 5 mm	POM-C	214.10
	IMP-08	-	SCEM 5 mm	POM-C	327.99
	IMP-09	-	SCEM 5 mm	POM-C	231.45
	IMP-10	-	SCEM 5 mm	POM-C	233
	IMP-11	-	SCEM 5 mm	POM-C	317.74
	IMP-12	-	SCEM 5 mm	POM-C	181.25
	IMP-01-A01	Cavities	SCEM 5 mm	POM-C	326.34
	IMP-01-A02	Cavities	SCEM 5 mm	POM-C	326.10
	IMP-01-A03	Cracks	SCEM 5 mm	POM-C	326.13
	IMP-01-A04	Chipped edge	SCEM 5 mm	POM-C	326.12

a SCEM: Solid Carbide End Mill (VHM in German); HSS: High-Speed Steel; Al 2007 T4: Aluminum alloy; POM-C: Polyoxymethylene copolymer; S235JR: Structural steel, t: cumulative cutting time of the tool (process time excluding rapid traverses and Z-movements).

The “*descriptive*” folder contains all metadata required to describe the experiments. This includes the list of tools used, the DoE and part descriptions. The DoE contains all technical details of each experiment. The part description includes all NC codes and CAD models for the respective parts, as well as CAD models of the material anomalies introduced in processes IMP-01-A01 to IMP-01-A04.

### 3.1 Data structure and signal overview

One key differentiator among process monitoring systems lies in the underlying data used for analysis. Therefore, a wide range of process signals were recorded during the experiments to maximize generalizability of the dataset. Acceleration and force data acquired via a DAC are provided as MATLAB timetable files (.mat) within the raw_data folder of each experiment. Additional process signals recorded using a SINUMERIK Edge are also included in the same folder as JSON files. These JSON files include both time series process signals (HFData) and the executed NC code (HFBlockEvent).

To facilitate initial analysis, as exemplified later in the article, all signals from the X, Y, and Z axes, as well as the spindle, were converted into CSV format and added to the processed_data folder. The JSON files were separated into three different CSVs:

• *header.csv* contains metadata for each recording,
• *hfblockevent.csv* contains the full machine-interpreted NC code, and
• *hfdata.csv* holds the aforementioned time series data.

In addition, all signal types were synchronized and combined into a single MATLAB timetable file. Alignment was based on a common start signal. To ensure temporal consistency, the 500 Hz Edge data were interpolated to 10 kHz using the PCHIP method (Piecewise Cubic Hermite Interpolating Polynomial), matching the sampling rate of the force and acceleration measurements. Table 2 provides an overview of all signal types recorded via the Edge system, along with their meaning and units. The signals recorded via the data acquisition tool are listed in Table 3.

**Table 2:** Signal types and description of the recording via the Siemens Industrial Edge system.2.

Signal	Description	Unit
CYCLE	Counter: incremented by one for each data point	-
ENC_POS	Encoder position: actual measured position	mm or degrees
DES_POS	Commanded position after fine interpolation	mm or degrees
CTRL_POS	Control position input after filter application	mm or degrees
CTRL_DIFF	Position deviation with DSC compensation	mm or degrees
CTRL_DIFF2	Position deviation without DSC compensation	mm or degrees
CONT_DEF	Contour deviation between calculated and actual path	mm or degrees
VEL_FFW	Velocity feedforward to the position controller	mm/s or deg/s
TORQUE_FFW	Torque feedforward to the drive system	Nm or N
CMD_SPEED	Commanded speed sent to the drive system	mm/s or deg/s
ENC1_POS	Current position of encoder 1	mm or degrees
ENC2_POS	Current position of encoder 1	mm or degrees
TORQUE	Calculated target torque/force for the drive	Nm or N
POWER	Electrical power consumption of the motor	W
CURRENT	Electrical current consumption of the drive	A
LOAD	Drive load: mechanical load on the motor	%

2 https://documentation.mindsphere.io/resources/html/manage-my-sinumerik-edge-app-publishing/en-US/developer-docu/sinumerikadapter.html.

**Table 3:** Signal types and description of the recording on the data acquisition tool.

Signal	Description	Unit
Time	Time elapsed since the start of recording	seconds
Sync_Signal	Periodic reference signal for alignment with Edge data	-
Force	Measured force in X, Y, and Z directions	N
Acceleration	Measured acceleration in X, Y, and Z directions	g

## 4 Experimental Design, Materials and Methods

The dataset was developed to provide a standardized foundation for validating intelligent process monitoring systems. While numerous monitoring approaches exist, the associated validation datasets are often heterogeneous and lack consistency in scope and documentation.

Process monitoring systems are commonly used for tasks such as anomaly detection [8,10,13], prediction of milling quality and stability [2,5,12,15,17], and tool condition monitoring [4,6,9]. Despite these different objectives, they all share the goal of autonomous quality improvement in industrial milling processes. Therefore, a common and comparable validation basis is crucial for their evaluation.

A review of existing validation datasets shows that many focus on simplified operations, such as pocket milling, and rarely include complex or functionally relevant parts. Only one of the reviewed studies used a fully functional workpiece as a validation object [3]. In many cases, the experimental setup is described only briefly, with limited information about the test parts themselves [2,12]. Visual representations of the validated parts are also uncommon, appearing in only about half of the reviewed works.

Moreover, geometries used in validation are often simple, and datasets typically contain data from only a single experiment or part. This limits the significance and transferability of results. Some researchers [8,9] rely on publicly available datasets such as the PHM 2010 Data Challenge [11], which were not designed with real-world milling validation in mind.

Publicly available datasets for milling process validation remain rare. In addition to the PHM 2010 Data Challenge, the NASA Milling Dataset is one of the very few open-access datasets in this domain. It focuses on the analysis of abrasive tool wear under controlled experimental conditions. Sixteen scenarios were tested, varying in depth of cut, feed rate, and material, with repeated runs until a predefined wear threshold was reached. However, the dataset lacks information on toolpaths and machined geometries. The use of a face mill suggests that straight linear milling operations were performed, with both tool entry and exit included in the recorded data. Signals include current measurements (DC and AC), as well as vibration and acoustic emission data recorded at the spindle and the machine table.

### 4.1 Conceptual design

To fulfill the previously mentioned requirements - especially a high degree of industrial realism combined with scientific rigor - the methodological framework of Design Science Research [7,14] was applied. The following outlines the methodical development of the three selected component types.

To ensure relevance, the first step involves identifying industrial sectors that are significantly impacted by agile production strategies. This helped define the scope of potential application scenarios, which include single-item production, variant production, and small-batch manufacturing. Consequently, the number of component types was limited to three, striking a balance between broad industrial coverage and a manageable, focused dataset.

To achieve a high degree of diversity, each (initially hypothetical) part was assigned different characteristics based on five key parameters (Table 4): material, size, complexity, milling strategy, and industrial sector.

**Table 4:** Assignment of three component types based on five key design parameters (morphological box).

Parameter	Variant 1	Variant 2	Variant 3
Material	Steel	Aluminum	Plastic
Size	Small	Medium	Large
Complexity	Simple	Medium	Complex
Strategy	Pocketing	Slotting	Contour milling
Sector	Prototypes	Consumer goods	Packaging

Based on these defined parameters, three concrete parts were developed:

- **Thermoforming mold (TF):** Milled from aluminum, this mold is designed for thermoforming thermoplastics used in packaging production. It features predominantly linear geometries.
- **Injection mold (IM):** Milled from steel, this mold is intended to produce housing for electronic components. It is characterized by its geometric complexity.
- **Impeller (IMP)**: A variety of impellers made from plastic were designed for small-batch production. These differ in blade count and geometry, representing high intra-part variation within a single category.

**Fig. 2:**
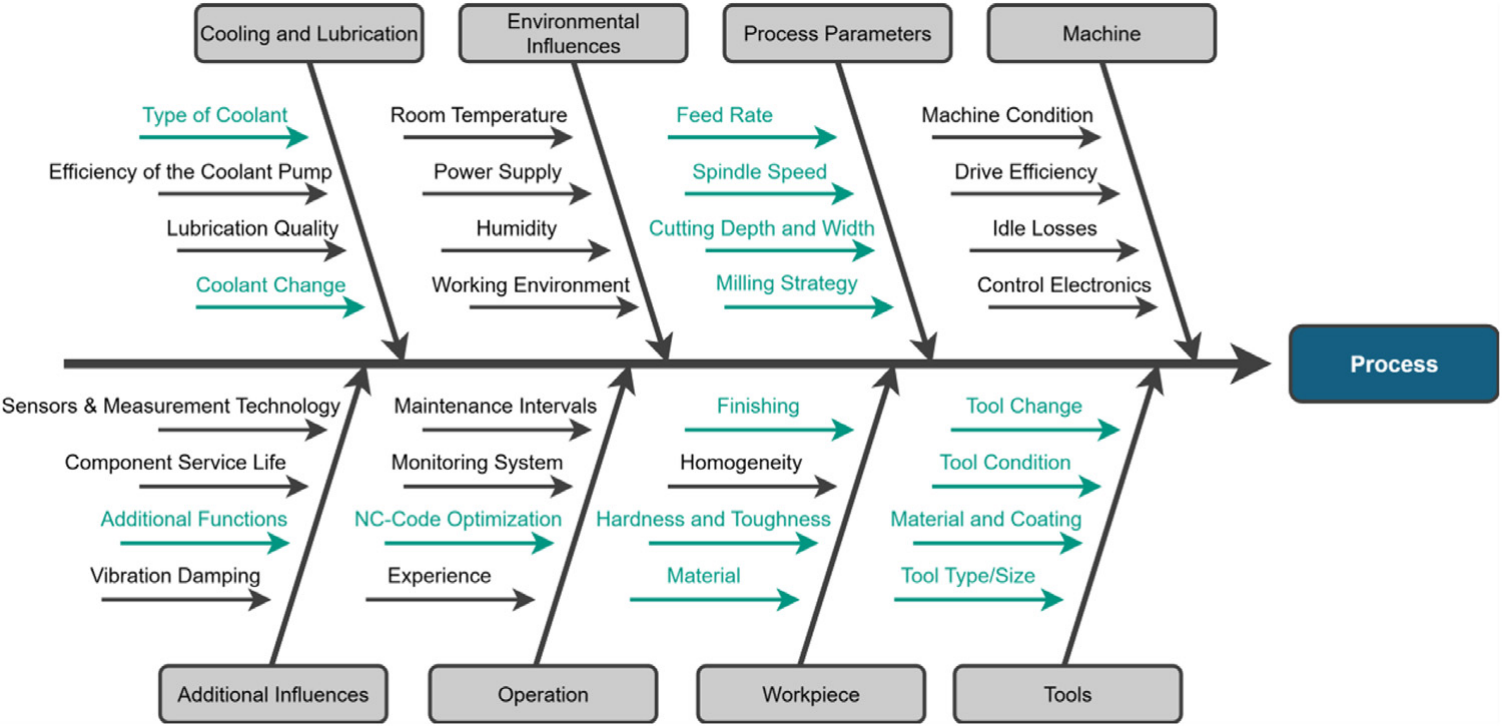
Selection of factors influencing the general milling process (leverage factors highlighted in green).

To finalize the experiments, so-called leverage factors were identified - i.e., controllable variables that influence the general milling process (see Fig. 2). Among these, the following were identified as relevant leverage factors: process parameters (e.g., feed rate, cutting speed), tool characteristics (e.g., geometry, coating), tool condition (e.g., new vs. worn), and workpiece material.

Additional features, such as cooling or lubrication, were deliberately excluded to keep the setup manageable and to enhance general applicability.

To systematically incorporate relevant anomalies, potential influences on milling quality were identified based on [1] and evaluated according to their feasibility, informativeness, and industrial relevance. Seven anomalies were then selected and categorized into three types of faults: those caused by the operator, the tool, or the workpiece. A broad spectrum of real-world anomalies is therefore covered.

To create a complete experimental plan, all selected leverage factors and anomalies were systematically assigned to the respective component types (see Table 1). This mapping is detailed in the corresponding DoE included in the dataset.

### 4.2 Components

This section provides an overview of the experiments conducted. For each component type standardized blocks with dimensions of 150 × 75 × 50 mm were used. These could be machined on both sides, with the stated dimensions representing the maximum allowable size. Smaller components, such as impellers, could be machined twice per side due to their compact geometry.

#### 4.2.1 Thermoforming mold

**Fig. 3:**
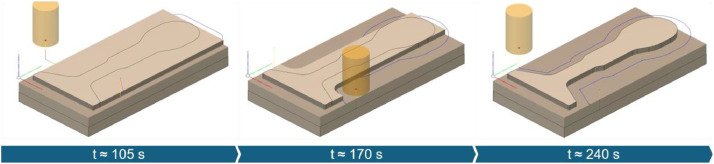
Toolpath strategy used for machining the thermoforming mold, shown here for ap = 5 mm, corresponding to TF-01.

The experiments for the thermoforming mold followed a structured plan aimed at examining different material removal rates. Toolpaths (as shown in Fig. 3) were segmented based on varying radial cutting depths. The series includes three processes with progressively increasing axial cutting depths.

Machining was carried out on aluminum blocks (Al 2007 T4) using a 20 mm HSS end mill. The focus of the thermoform was to achieve efficient machining, considering both tool wear and process anomalies. Accordingly, the depth of cut and, in later runs, the cutting speed were increased to provoke potential machine overload (TF-03-A01 & TF-03-A02). Additionally, an operator error was simulated by inadvertently increasing the feed rate (TF-01-A01). To study tool wear, adhesive wear mechanisms typical of aluminum were introduced. Therefore, the cutting speed was reduced at a medium depth of cut to give an evaluation option for a monitoring system's ability to detect the formation of built-up edges (TF-02-A01).

#### 4.2.2 Injection mold

**Fig. 4:**
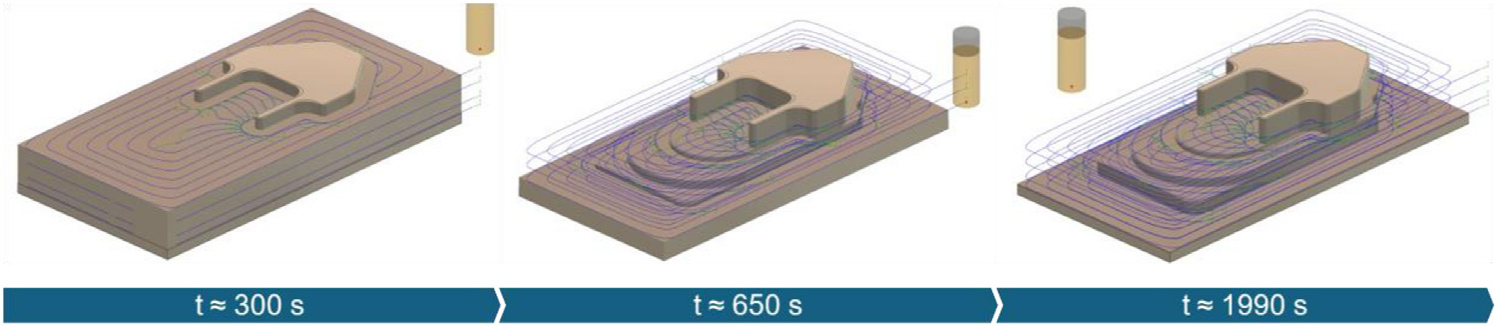
Toolpath strategy used for roughing the injection mold (time (t) corresponding to IM-01R).

**Fig. 5:**
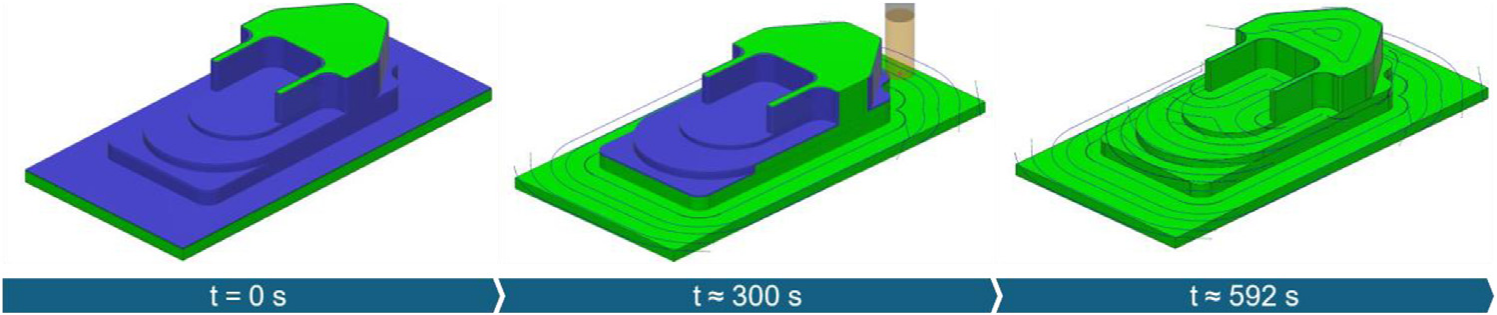
Toolpath strategy used for finishing the injection mold (blue areas indicate stock remaining after roughing; time (t) corresponding to IM-01F).

The injection mold was designed to represent high geometric complexity. To further increase process variability, a pocket feature was added. The chosen process strategy included both roughing (Fig. 4) and finishing (Fig. 5) operations. A stock allowance of 0.5 mm was left during roughing, which was then removed in the finishing pass.

Experiments were performed on steel blocks (S235 JR) using a 10 mm solid carbide end mill. To simulate material anomalies, two blocks were modified with axial and radial boreholes of varying diameters (IM-01R-A01 & IM-01R-A02), enabling reproducible emulation of shrinkage cavities (blowholes). In addition, progressive tool wear was introduced and tracked across six successive roughing and finishing passes (IM-01R-A01 to IM-01R-A05), with wear quantified via the cutting time of the tool. The high hardness of the material made it well-suited for simulating abrasive wear.

The finishing operations were performed without prior face milling, resulting in variable surface conditions. These inconsistencies represent anomalies (IM-01F-A01). In another experiment, chatter was induced by doubling the feed rate during a finishing operation (IM-02F-A01).

Overall, the anomalies in this series comprise material defects, process-related disturbances, and tool-related issues.

#### 4.2.3 Impeller

**Fig. 6:**
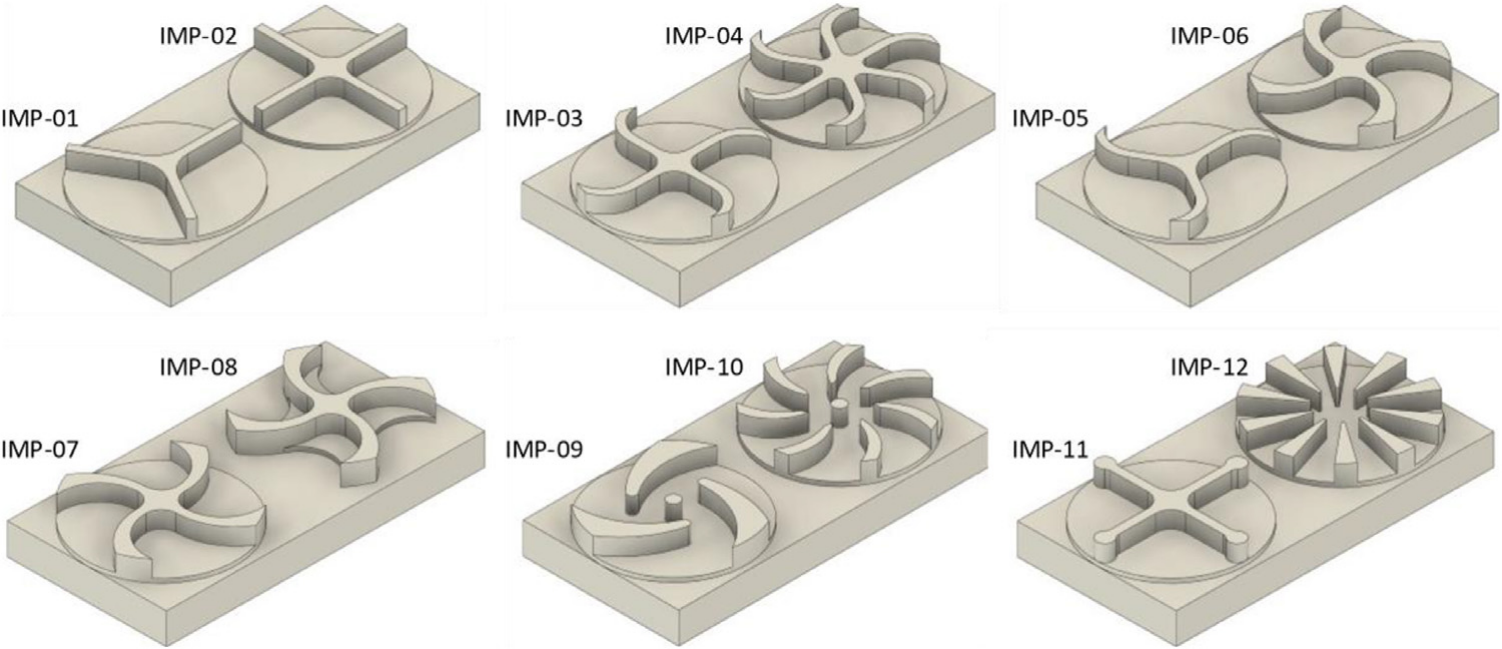
All designed variations of the impeller geometries.

**Fig. 7:**
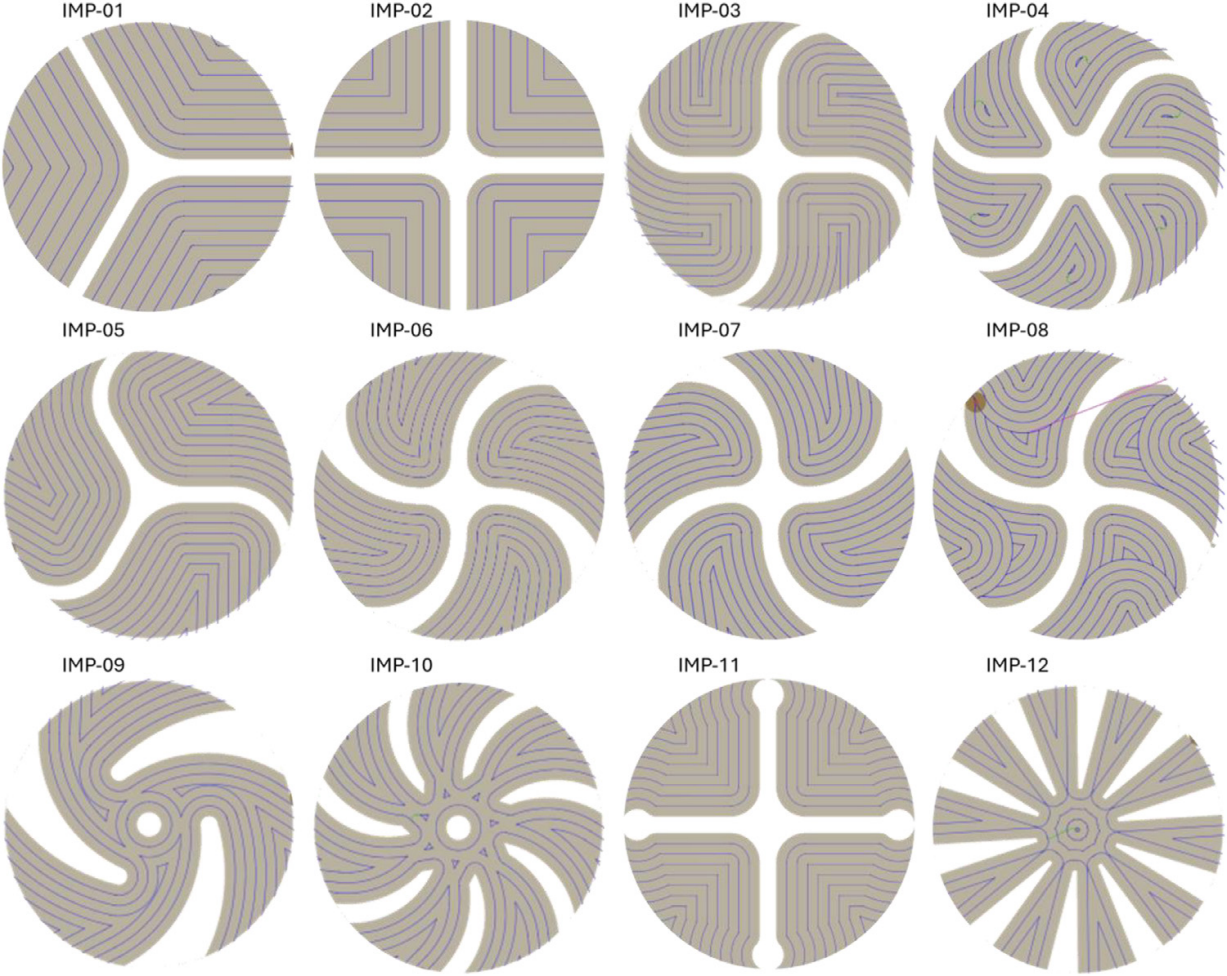
Machining strategies used across the twelve impeller variants.

Twelve impellers with different geometries (Fig. 6) were modeled for the experiments. These vary in terms of blade shape and count (Fig. 7). Due to their compact size, two impellers could be machined side by side on a single block. Before machining, a base contour was milled to create two raised circular pads, serving as the starting point for further machining.

**Fig. 8:**
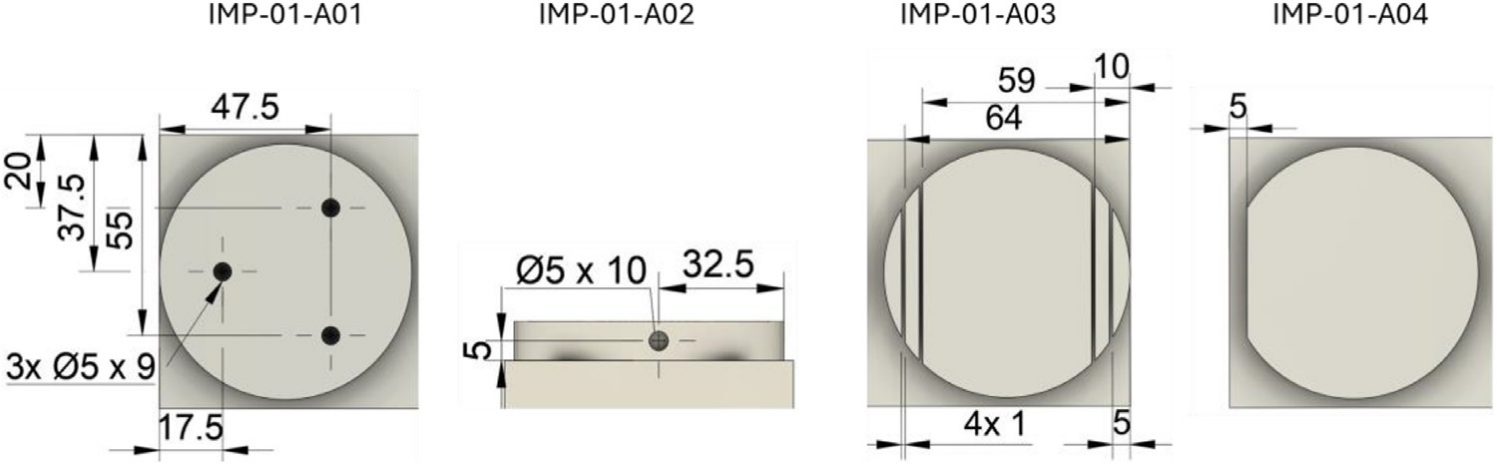
Material anomalies introduced into the base contours of IMP-01-A01 to IMP-01-A04.

The impellers were milled from plastic blocks (POM-C) using a 5 mm solid carbide end mill. Four types of defects (cracks and cavities) were added to the base contours to generate material anomalies (Fig. 8). These anomalies were all applied to the geometry of the first impeller (IMP-01). Crack formation during machining is a known issue in softer materials like POM-C and is often influenced by batch-specific properties.

### 4.3 Experiment execution

All components were modeled using a CAD program (Autodesk Fusion 360) to implement the experimental plan outlined above. The associated CAM environment was used to generate the required toolpaths, which were then post-processed into NC code.

A synchronization command was inserted at the beginning of the NC code to synchronize the data recorded by the edge device and the DAC. This step was necessary because both recordings were manually started before the machining process began.

**Fig. 9:**
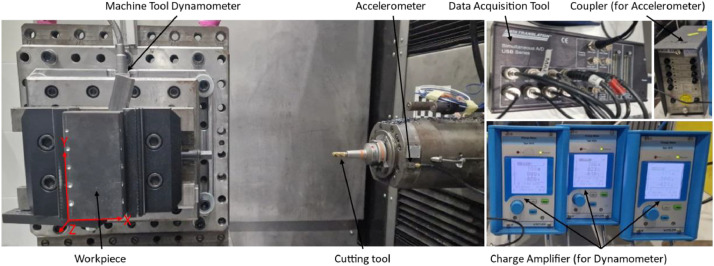
Overview of the experimental setup and sensor placement.

All experiments were conducted according to the DoE. Internal process signals - including current and position data - were recorded via the edge device at a sampling rate of 500 Hz. In addition, force and acceleration data were measured using respective sensors at 10 kHz. These signals were recorded in volts, amplified using charge meters, and saved to a computer via a DAC. Calibration of the signals was performed in MATLAB. The experimental setup can be seen in Fig. 9.

### 4.4 Data preparation

The Edge stores recorded data in zipped JSON files with a maximum size of 100 MB per file. Therefore, longer recordings are stored in subsequent JSON files, each of which is assigned a unique UUID. Each JSON consists of a header with machine metadata and a payload section, which contains both the executed NC code (HFBlockEvent) and the time series data (HFData). Data were recorded for six axes: X, Y, Z, tool changer, B-axis, and main spindle - numbered in that order. For preprocessing, only the signals from the X, Y, and Z axes, as well as the main spindle, were converted into CSV format (*hfdata.csv)*.

The raw MATLAB timetable files contain acceleration and force data in X, Y, and Z directions, which are consistently defined relative to the tool coordinate system. As described above, the raw data from the Edge and the DAC were synchronized and combined into an additional unified MATLAB timetable.

## 5 Exemplary Evaluation

**Fig. 10:**
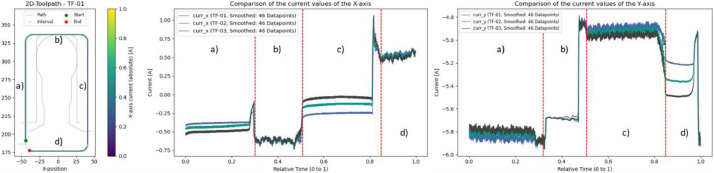
Evaluation of varying radial (colors: blue = 5 mm, green = 10 mm, black = 15 mm) and radial cutting depths (a and c: 10 mm, b: 5 mm, d: 0 mm). Center: Smoothed current values of the X-axis. Right: Smoothed current values of the Y-axis.

**Fig. 11:**
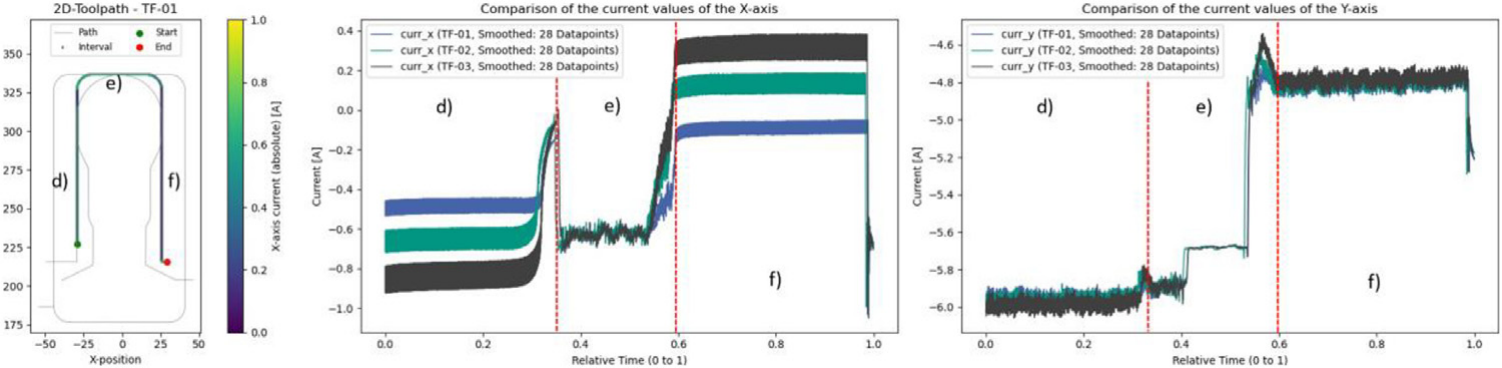
Evaluation of varying axial (colors: blue = 5 mm, green = 10 mm, black = 15 mm) and radial cutting depths (d and f: 15 mm, e: 0 mm). Center: Smoothed current values of the X-axis. Right: Smoothed current values of the Y-axis.

For general observations of different process variables, reference experiments without anomalies are especially suitable. These runs allow a systematic examination of the influence of varying widths and depths of cut. Some exemplary results are shown in Fig. 10 and Fig. 11.

Interestingly, higher material removal rates do not always result in increased current values. In Fig. 10, the plot for the Y-axis (with a radial depth of cut of ae = 10 mm) shows that a larger axial depth of cut (ap = 15 mm; gray) leads to lower current values in areas a) and c) compared to a smaller axial depth of cut (ap = 5 mm; blue). This effect is offset in Fig. 11, where a greater radial depth of cut (ae = 15 mm) causes the expected increase in current in areas d) and f) when the axial depth of cut is increased.

The effects of the implemented anomalies on the process signals are of particular interest for evaluating process monitoring systems. The following sections present selected anomalies and their representative evaluations.

### 5.1 Thermoforming mold: adhesive wear (TF-02-A01)

**Fig. 12:**
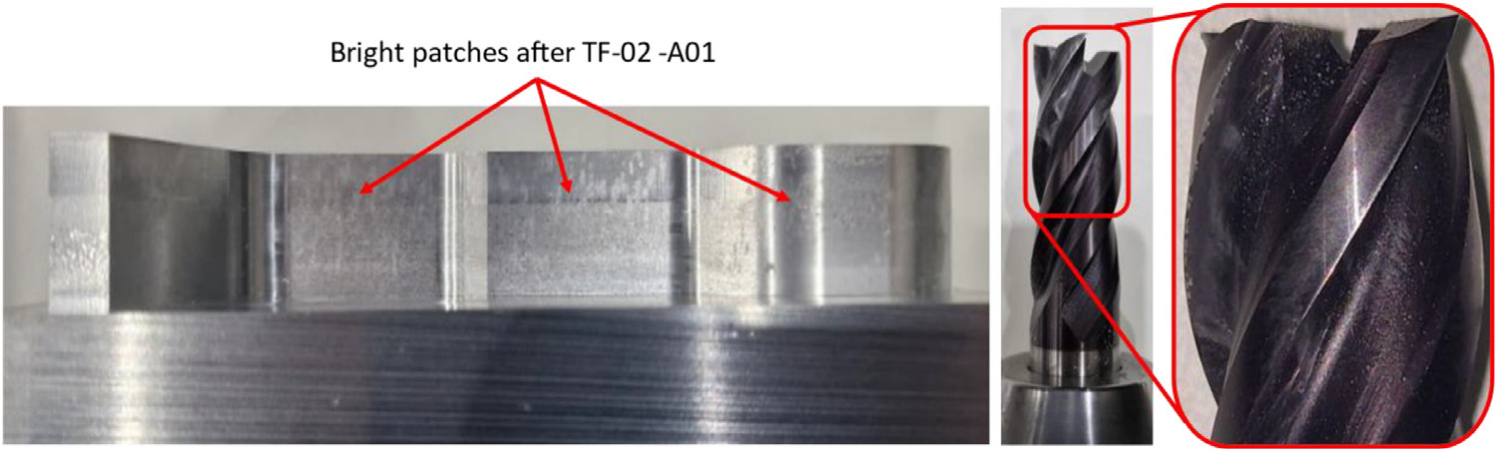
Left: Bright patches visible on the surface after TF-02-A01. Right: Built-up edge formation on the tool.

**Fig. 13:**
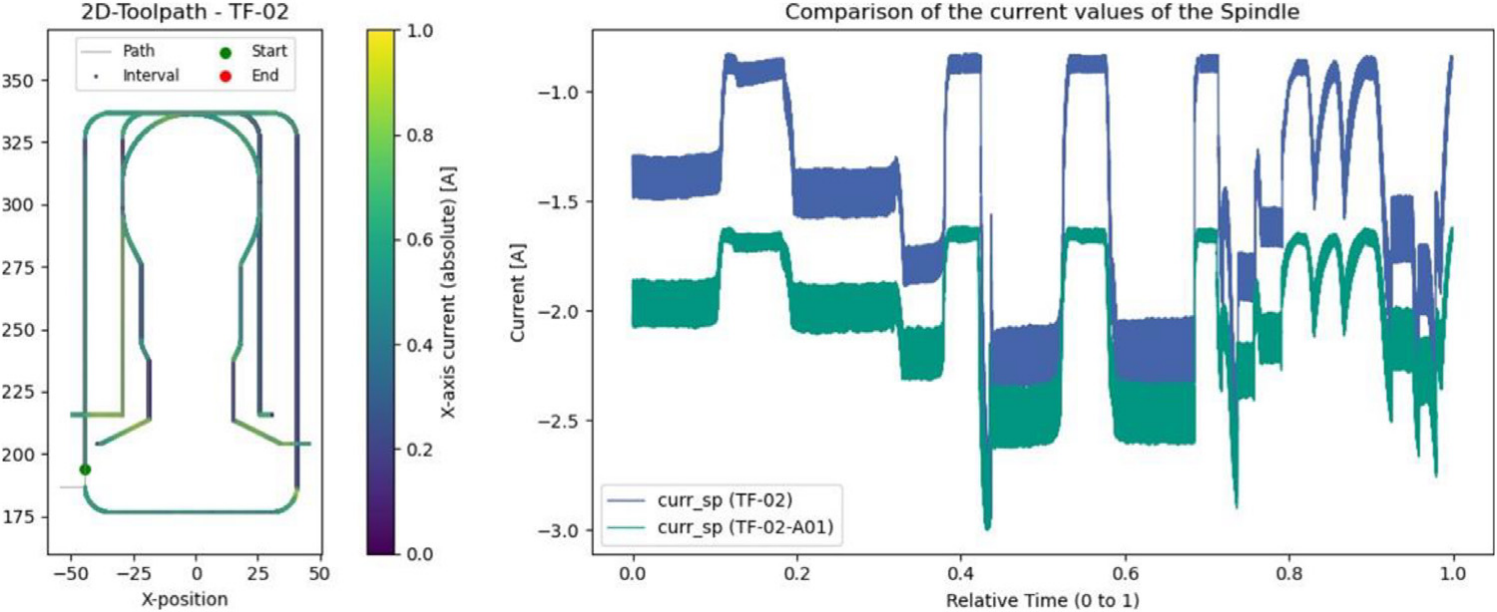
Left: Toolpath and evaluated interval. Right: Differences in spindle current between TF-02 (blue) and TF-02-A01 (green) with increasing built-up edge formation for this section.

A built-up edge was observed on the tool after completing experiment TF-02-A01. Bright patches were also visible on the vertical surfaces of the machined part (Fig. 12). During the trials conducted at reduced cutting speed, the spindle current values (Fig. 13) were notably higher - likely due to increased forces caused by lower feed rates and spindle speeds. The onset of the built-up edge did not occur at the very beginning of the process.

### 5.2 Thermoforming mold: overload (TF-03-A01 & TF-03-A02)

**Fig. 14:**
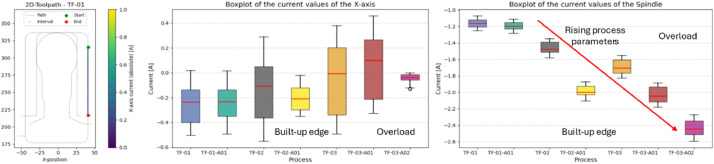
Distribution of current values from all thermoforming mold experiments during linear feed in the Y-direction. Left: Toolpath with evaluated interval. Center: X-axis current values for this interval. Right: Spindel current values for this interval.

In addition to overall comparisons, it can also be useful to analyze specific sections of the toolpath. For example, during a cutting operation in the negative Y-direction, it was observed that current fluctuations on the X-axis were particularly low in experiments TF-03-A01 and TF-03-A02 (Fig. 14). This is noteworthy because these two experiments differ significantly in terms of process parameters, while runs with intermediate settings showed greater variation. This behavior may be explained by exceeding the machine’s stability threshold in these cases.

The expected increase in spindle current is also evident in the same segment. Only TF-02-A01 - affected by built-up edge formation - deviates slightly from this trend, with a more rapid increase in current. This phenomenon was already observed in the earlier comparison between TF-02 and TF-02-A01.

### 5.3 Injection mold: chatter (IM-02F-A01)

**Fig. 15:**
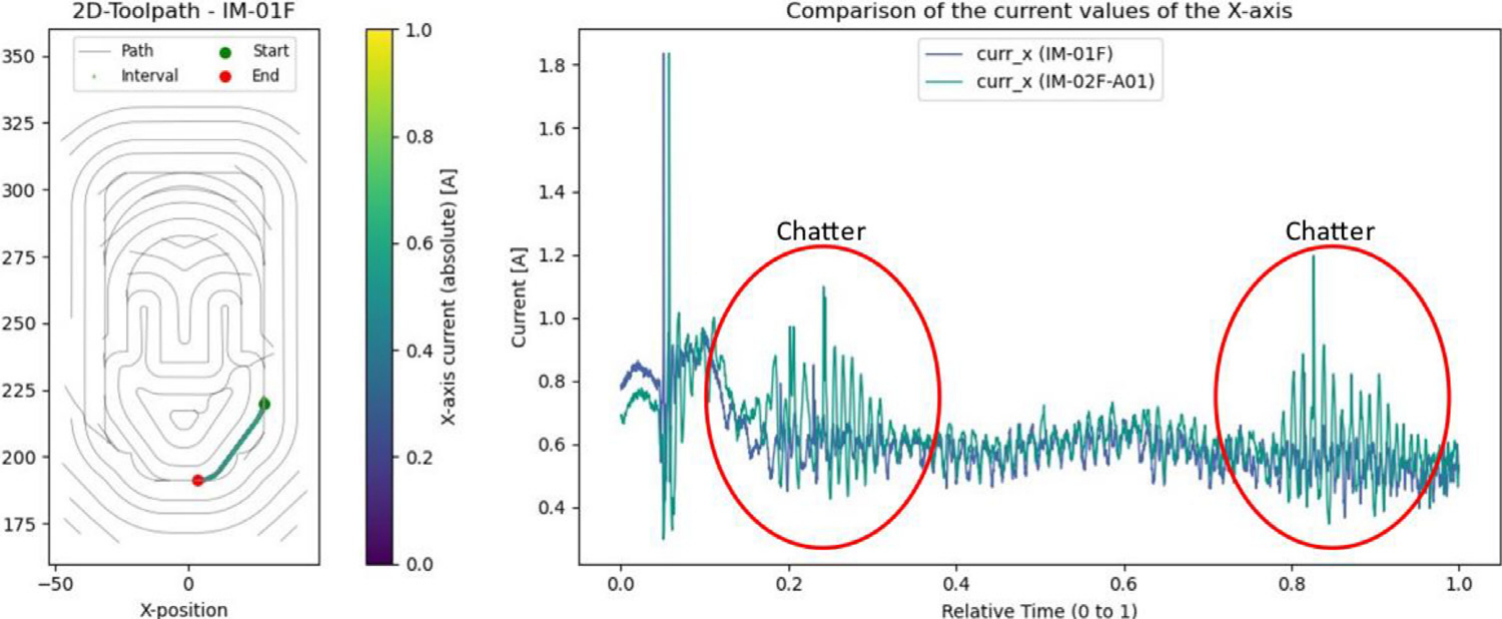
Left: Toolpath and evaluated interval. Right: X-axis current value with occurrence of chatter in trial IM-02F-A01 (green) compared to the reference trial (blue) for this interval.

**Fig. 16:**
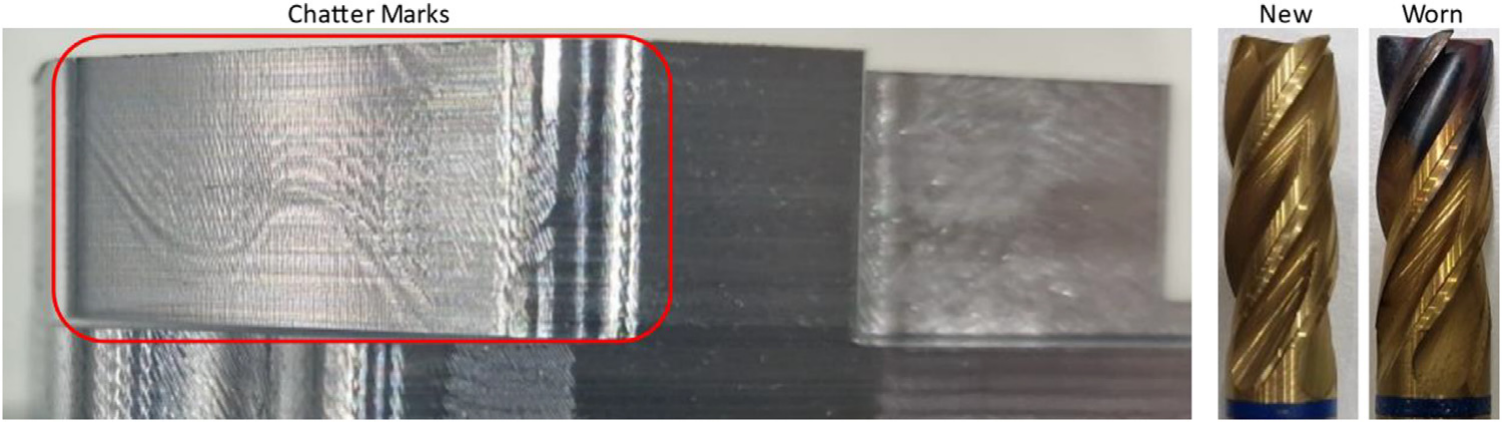
Left: Chatter marks on the workpiece in trial IM-02F-A01. Right: Tool wear after completion of all six wear tests on the injection mold.

Another example of a process anomaly is chatter, which was deliberately introduced during a finishing operation by doubling the feed rate. This effect is of particular interest for process monitoring systems focused on predicting milling stability. Chatter is clearly visible in the X-axis current signals (highlighted in Fig. 15). It is also evident from the resulting surface of the machined part as shown in Fig. 16.

### 5.4 Injection mold: blowholes (IM-01R-A01 & IM-01R-A02) and abrasive wear

**Fig. 17:**
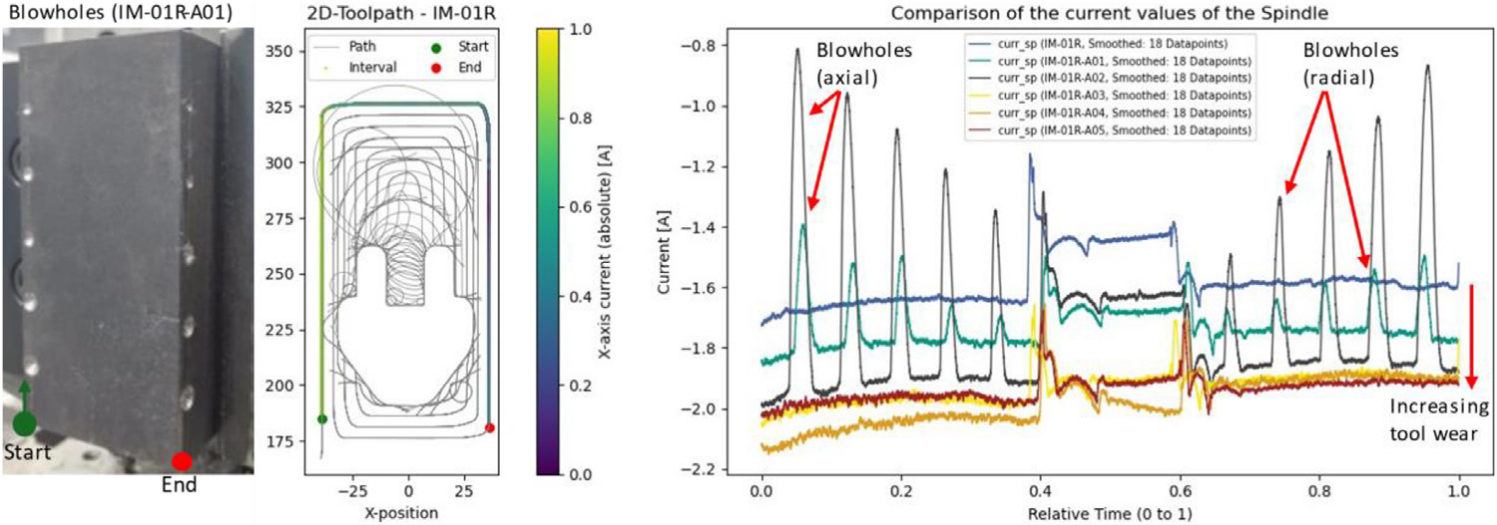
Left: Workpiece for trial IM-01R-A01 with blowholes. Center: Evaluated time interval. Left: Spindle current over all six roughing passes on the injection mold.

For anomaly detection applications, the artificially introduced cavities in the steel blocks offer useful validation scenarios. Ten holes - five axial and five radial - were drilled into each block (see Fig. 17). The holes in IM-01R-A01 were 1 mm deep, and those in IM-01R-A02 were 5 mm. These blowholes are clearly reflected in the spindle current signals, which show significant drops at the corresponding toolpath segments.

The gradual progression of tool wear across all six roughing cycles is also clearly observable. As expected, spindle current increases over time, although the magnitude of the difference between cycles diminishes as wear progresses.

### 5.5 Injection mold: irregular stock allowance (IM-01F-A01)

**Fig. 18:**
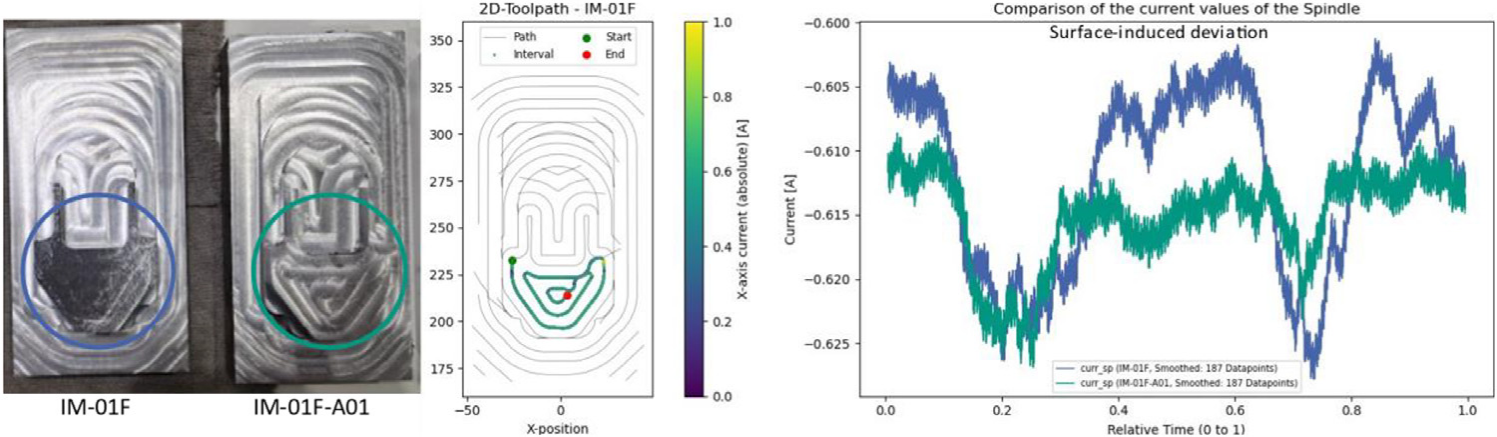
Comparison of different raw part allowances during the finishing of the injection mold. Left: Variation in material removal on the workpiece. Right: Differences in spindle current between IM-01F (blue) and IM-01F-A01 (green).

The steel blocks were not face-milled before finishing, resulting in uneven starting surfaces. This is especially relevant for finishing operations, where the tool is expected to skim the surface without significant material removal. Among all trials, experiment IM-01F (shown in blue in Fig. 18) comes closer to this ideal condition than IM-01F-A01 (shown in green).

### 5.6 Impeller: cavities (IMP-01-A01 & IMP-01-A02)

**Fig. 19:**
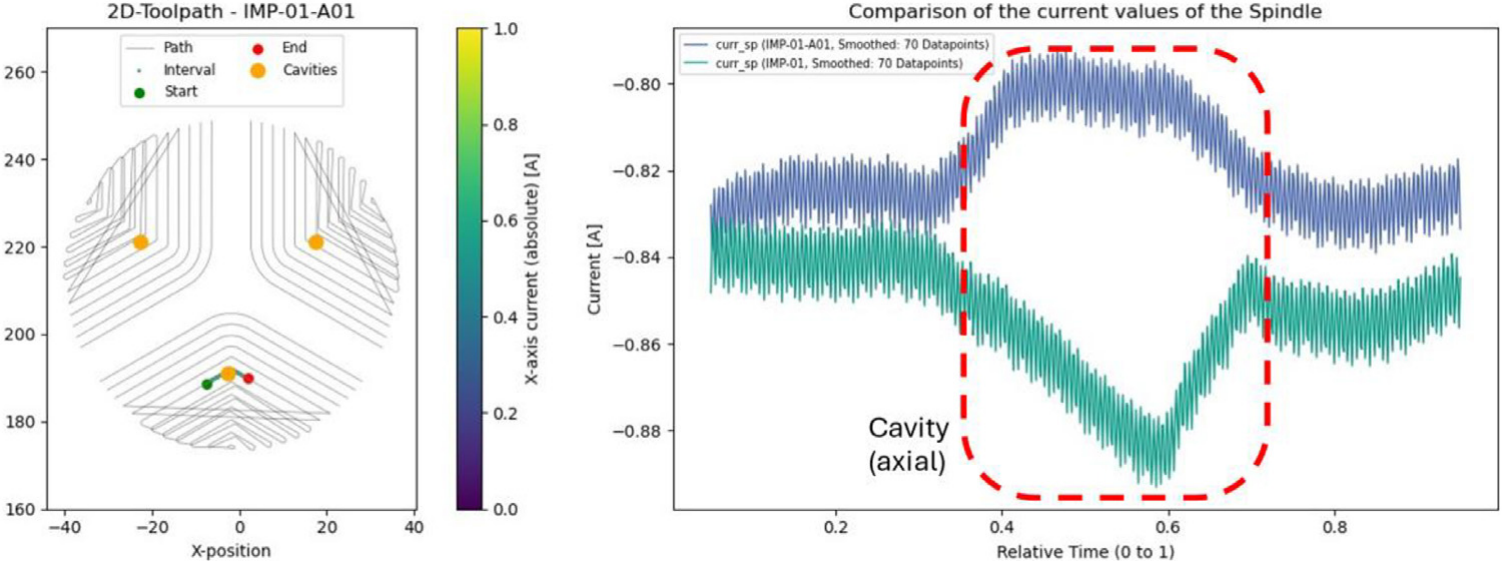
Left: Evaluated interval and position of the introduced axial cavities. Right: Comparison of spindle current between IMP-01 (green) and IMP-01-A01 (blue) for this interval.

**Fig. 20:**
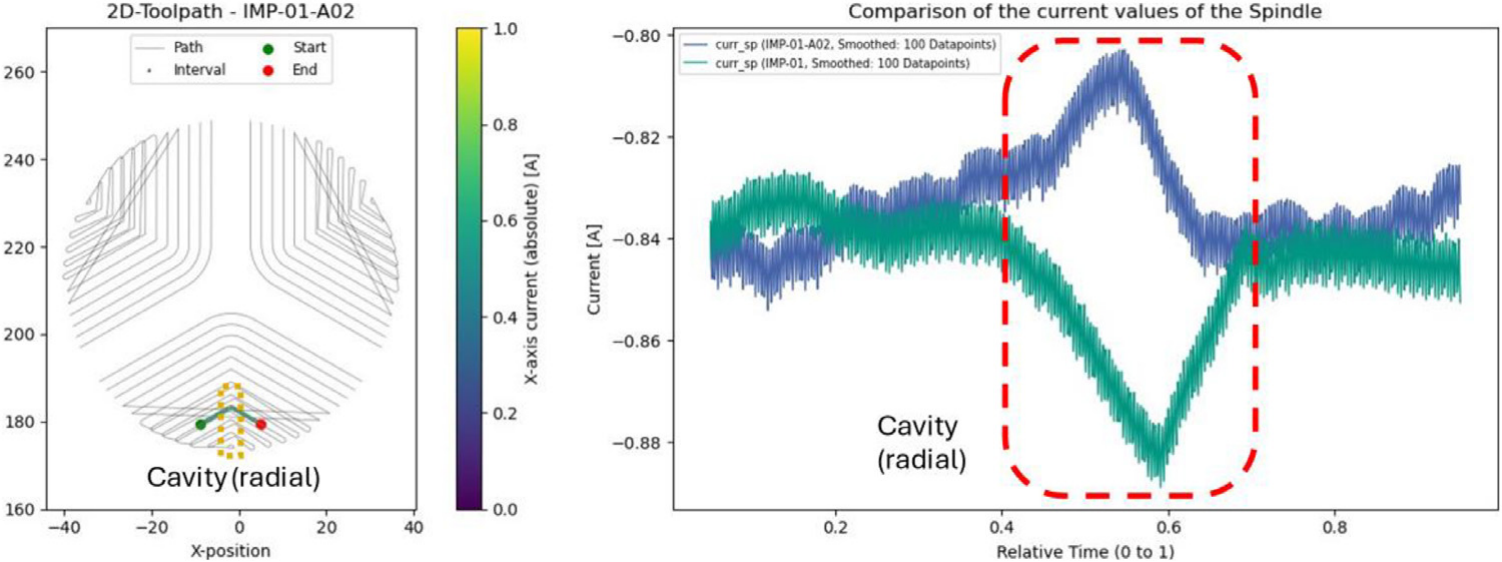
Left: Evaluated interval and position of the introduced radial cavity. Right: Comparison of spindle current between IMP-01 (green) and IMP-01-A02 (blue) for this interval.

Axial and radial boreholes were introduced in two impellers prior to machining. These anomalies produced noticeable drops in spindle current at specific locations, consistent with the presence of the cavities (as seen in Fig. 19 for axial and Fig. 20 for radial cavities). Further analysis could investigate how different toolpath strategies interact with such material defects.

### 5.7 Impeller: cracks and chipped edge (IMP-01-A03 & IMP-01-A04)

**Fig. 21:**
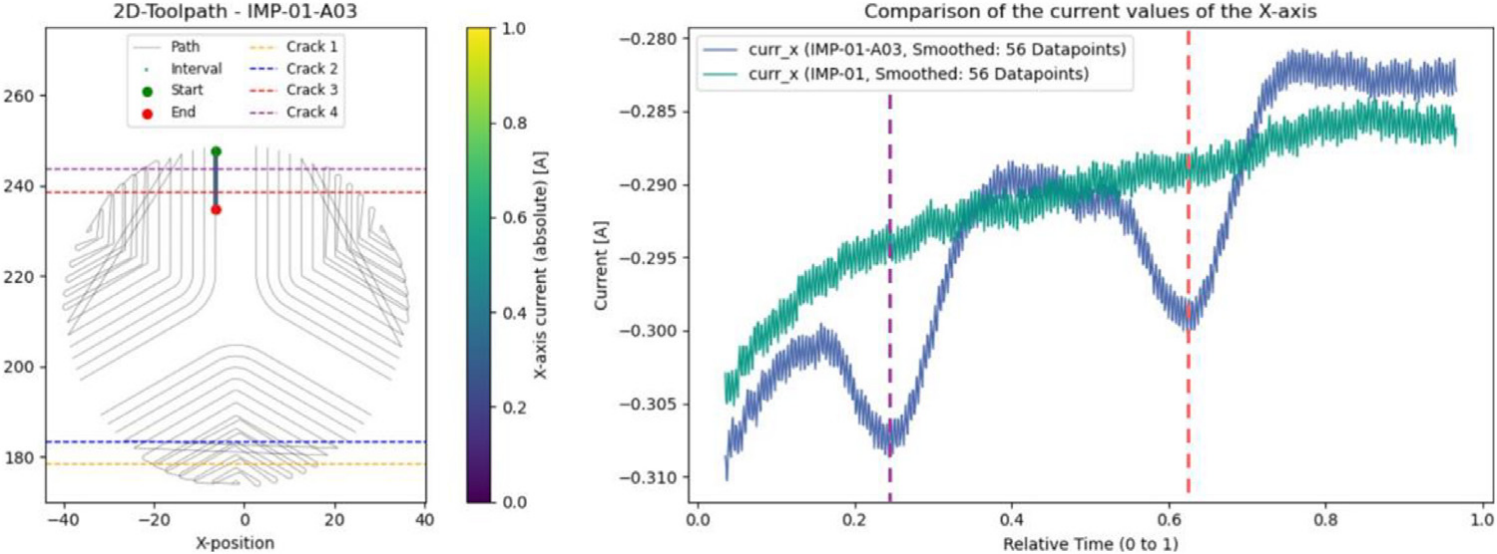
Left: Evaluated interval and position of the introduced cracks. Right: Comparison of X-axis current between IMP-01 (green) and IMP-01-A03 (blue) for this interval.

For a more detailed analysis, subtle cracks were introduced into another impeller. Their effect is visible in both the spindle and X-axis current signals, as shown in Fig. 21 for the X-axis. In the blue plot, the cracks cause clear, momentary current drops as the tool passes through the damaged regions, covering an interval of about 1.5 s.

**Fig. 22:**
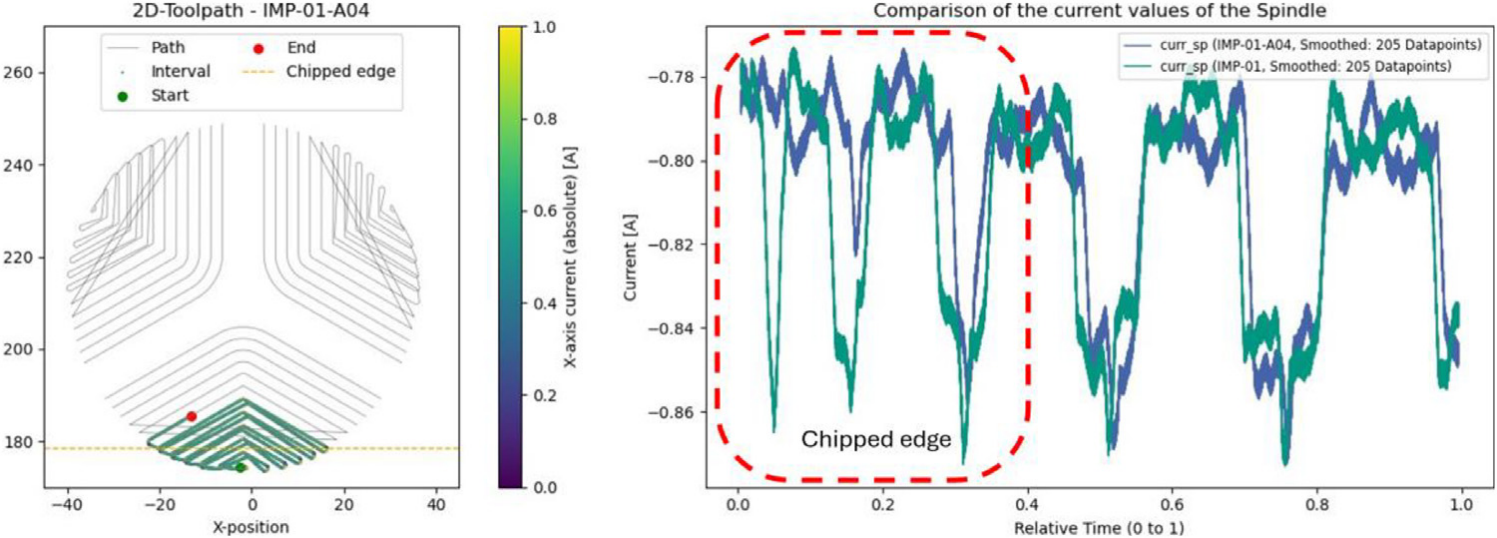
Evaluated interval and position of the chipped edge. Right: Comparison of spindle current between IMP-01 (green) and IMP-01-A04 (blue) for this interval.

In a separate trial (IMP-01-A04), an edge of the impeller was deliberately broken off before machining. This defect is visible at the beginning of the process as significantly lower current peaks (see Fig. 22). As the tool moves beyond the missing edge, the signal normalizes.

## 6 Application

**Fig. 23:**
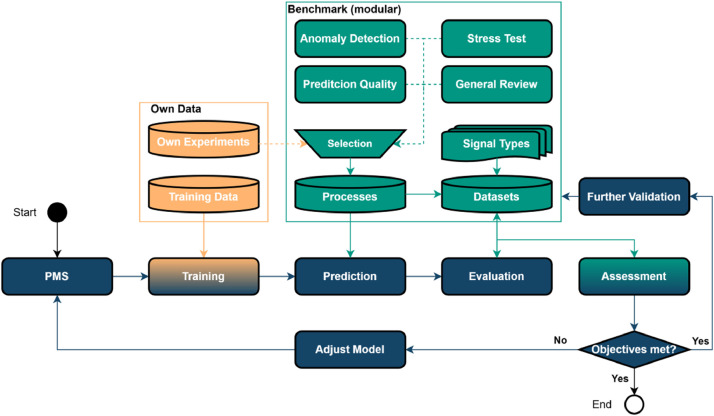
Possible data pipeline using the dataset for validation of the PMS (Process Monitoring System).

The dataset can be used in two primary application scenarios. First, it enables researchers to gain deeper insights into the performance of their process monitoring systems. Users can also integrate their own data into the dataset to analyze specific process segments in a targeted manner. A possible data pipeline illustrating this application is presented in Fig. 23.

A possible approach would be to start by evaluating more prominent anomalies, such as cavities, to establish a baseline validation. The system can then be refined by analyzing more subtle anomalies, such as progressive tool wear. Validating monitoring systems with the use of this dataset allows researchers to publish meaningful, comparable, and reliable performance metrics. At the same time, the effort required to develop and document a custom validation dataset is eliminated. The standardized benchmark also enables researchers to highlight the specific strengths and weaknesses of their systems.

**Fig. 24:**
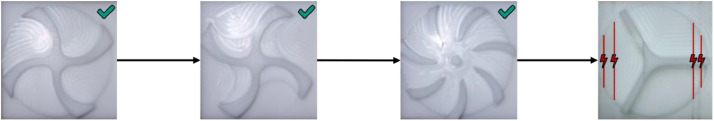
An exemplary use case for the application of the dataset is the monitoring of the production of a pump prototype.

From an industrial perspective, the dataset offers broad applicability in agile manufacturing contexts. The included impellers not only represent traditional impeller designs but also generalize to a wide range of rotating components, such as gears, turbine wheels, and similar parts. These components are common across many industries, including automotive, mechanical engineering, aerospace, and energy. Manufacturers of custom components, such as pump manufacturers, can thus gain valuable insights into the capabilities of intelligent process monitoring systems tailored to their needs. Such an application is illustrated in Fig. 24.

The dataset demonstrates the predictive potential of monitoring systems in individualized manufacturing. It enables evaluation of how well such systems can adapt to different part geometries and whether they are capable of detecting inhomogeneities in new components, especially in agile environments, where no reference values from identical parts are available, but only empirical data from similar prior processes. Additionally, the dataset supports assessment of active data management functions, such as monitoring for overfitting behavior within the system.

## Limitation

The dataset inherently reflects machine-specific characteristics that influenced the experiments. Most notably, the machine’s stability thresholds are highly dependent on its mechanical properties. Since the milling machine used features a closed-loop control system, this directly affected process variables such as current consumption. Therefore, it is not feasible to reproduce the dataset identically on a different machine. To address the effects specific to individual machines, researchers could consider normalization or domain adaptation techniques to improve the transferability of models developed using this dataset.^1^

Another challenge relates to the controlled induction of process anomalies. In contrast to material defects, process-related anomalies cannot always be introduced in a fully repeatable manner. Additionally, overlapping effects - such as multiple forms of tool wear- are unavoidable.

It is important to note that the accelerometer was mounted near the spindle, which never runs perfectly concentric. These mechanical irregularities directly influence the acceleration signals. Likewise, electromagnetic interference may affect current measurements. Such disturbances cannot be entirely avoided but reflect the realities of actual production environments.

While the dataset offers a broad basis of realistic manufacturing conditions, it is neither feasible nor desirable to cover every possible scenario and anomaly within a single dataset. Excessive expansion would reduce clarity and comparability. Instead, the modular nature of the benchmark allows users to tailor and extend the dataset to suit their own research or application needs.

## Ethics Statement

The authors confirm that they have read and followed the ethical requirements for publication in Data in Brief. The current work does not involve human participants, animal experiments, or any data collected from social media platforms.

## Credit Author Statement

**Robin Ströbel:** Conceptualization, Methodology, Data Curation, Formal analysis, Writing - Original Draft. **Maximilian Kuck:** Conceptualization, Methodology, Data Curation, Formal analysis, Visualization, Writing - Original Draft. **Florian Oexle:** Data Curation, Writing - Review & Editing. **Hafez Kader:** Writing - Review & Editing. **Alexander Puchta:** Writing - Review & Editing, Supervision. **Benjamin Noack:** Supervision. **Jürgen Fleischer:** Supervision.

## Data Availability

KITopenA Multimodal Dataset for Process Monitoring and Anomaly Detection in Industrial CNC Milling (Original data)

## Footnotes

1 A complementary dataset (DOI: 10.35097/vnnu3n9z7ndsnhfd) comprising partially overlapping experiments recorded on a second machine (CMX 600V from DMG Mori) is available for cross-machine validation.
